# Aging Reprograms the Signaling, Metabolic, and Gene Regulatory Dynamics in Murine Bone Marrow-Derived Mesenchymal Stem Cells

**DOI:** 10.3390/app16083779

**Published:** 2026-04-13

**Authors:** Md Tamzid Hossain Tanim, Aarushi Patel, Venu Pandit, Luke Fracek, Anja Nohe

**Affiliations:** Department of Biological Sciences, College of Arts and Sciences, University of Delaware, Newark, DE 19716, USA

**Keywords:** bone marrow mesenchymal stem cell, BMSC, aging, signaling, cell fate, phosphoproteomics, proteomics

## Abstract

Bone marrow-derived mesenchymal stem cells (BMSCs), owing to their osteoblastogenic differentiation potential, are crucial for maintaining bone homeostasis and remodeling. Nevertheless, in aging and age-related bone diseases like osteoporosis, BMSCs show significantly diminished osteogenic potential, with a concomitant increase in adipogenic differentiation. The aged BMSCs also become desensitized to BMP2 stimulation to a large extent and exhibit aberrations in BMP2 signaling. However, the molecular mechanisms facilitating this shift in lineage commitment and mediating the cellular dysfunctions remain elusive. This knowledge gap hinders the development of regenerative strategies for skeletal aging and osteoporosis. This study employed an integrative tandem mass tag (TMT)-based phosphoproteomic and total proteomic profiling on BMSCs isolated from young (6-month) and aged (15-month) C57BL/6 (B6) mice to elucidate global alterations in both protein activity and expression. The analysis identified more than 500 proteins that underwent significant alterations (BH-adjusted *p*-value < 0.05) either in phosphorylation or expression between young and aged BMSCs. Many lineage-specific markers also underwent changes in both phosphorylation and expression with aging. Additionally, key biological processes, including cellular metabolism, clathrin-mediated endocytosis, and nucleocytoplasmic transport mechanisms, were enriched for the deregulated proteins. Signaling proteins, ERK-1/2, had increased activating phosphorylation in the aged BMSCs, while transcription factors Lrrfip1, Ruvbl1, and Ruvbl2 also exhibited dysregulated activity and abundance in the aged BMSCs. The findings from the study adds significant mechanistic insights into how aging disrupts signal transduction, metabolism, and transcriptional program in BMSCs, contributing to age-associated loss of bone mass and reduced skeletal regenerative capabilities. Through the identification of key mediators of BMSC dysfunction seen in aging, this work offers a strong foundation in devising potential therapeutic strategies to restore diminished osteogenic potential and treat osteoporosis.

## 1. Introduction

Mesenchymal stem cells (MSCs) are a unique type of multipotent stem cells derived from mesoderm and can be found in adult humans [1]. MSCs are primarily found in adult bone marrow and adipose tissues’ stromal vascular fraction [2]. They have also been found in placenta and umbilical cord tissue, which are disposed of at birth [2]. Bone-marrow-derived MSCs (BMSCs) are a key MSC population found in adult humans. They have the potential to differentiate into a wide variety of cell lineages, including, but not limited to, osteoblasts, adipocytes, chondrocytes, neural lineage cells, fibroblasts, and myocytes [3]. These cells account for only 0.001–0.01% of the entire bone marrow microenvironment’s cell population [4]. BMSCs show a great degree of heterogeneity, with many individual subtypes exhibiting distinct cellular phenotypes and functions [5]. Each subpopulation of cells has a unique morphology with different proliferative and differentiation potential [5]. BMSCs express MSC markers like CD73, CD90, and CD105, in addition to markers like STRO-1 and Stage-specific embryonic antigen 1 (SSEA) [6].

A plethora of regulatory factors, including extracellular and intracellular signaling molecules and transcription regulatory proteins, are involved in the fate decision of BMSCs. Osterix (Transcription Factor Sp7), Runx2 (Runt-related transcription factor 2), and Dlx5 (Distal-less homeobox 5) are the primary transcription factors that drive the osteoblastic fate of BMSCs [7]. During the chondrogenic differentiation of BMSCs, extracellular signal molecules like TGF-β1 (Transforming growth factor β1), BMP2 (Bone morphogenetic protein 2), and BMP4 (Bone morphogenetic protein 4) stimulate the activation/expression of key transcription factor Sox9 (SRY-box transcription factor 9), which then promotes the chondrogenic fate of BMSCs [8,9]. During adipogenic fate decision, two master regulators of transcription, namely, PPARγ (Peroxisome proliferator-activated receptor gamma) and C/EBPα (CCAAT/enhancer-binding protein alpha), induce the expression of adipogenic genes in response to different extracellular stimuli, including BMP4 (in C3H10T1/2 cells), as well as low concentrations of BMP2 [10].

BMSCs are particularly important for bone homeostasis and remodeling due to their osteogenic potential [11]. During bone homeostasis, BMSCs undergo osteogenic differentiation, producing osteoblasts, which then mature into osteocytes, and eventually aid in bone formation [12]. BMSCs also contribute to bone remodeling by providing the osteoblastic cells that help regenerate bone tissue after damage or during growth. They also play an important role in repairing fractures within the bone. Bone fracture or injury initiates a series of cellular and molecular pathways that regulate BMSCs’ activity, leading to fracture healing and bone and cartilage remodeling, reestablishing skeletal integrity [13,14]. Their lineage commitment is influenced by various molecular signals within the bone marrow microenvironment, including growth factors and cytokines [15–17]. Understanding the regulation of BMSC differentiation and their role in bone homeostasis is crucial for developing solutions to bone and joint conditions and disorders, including osteoporosis (OP) [18–20]. OP currently affects around 10 million Americans, severely disrupting the quality of life, while another 18 million people are at risk of developing it at a later stage of life [21]. As options for OP treatments remain limited, it is crucial to study BMSCs to determine how we can influence cell fate decisions.

Aging significantly affects BMSC function, leading to a decline in bone formation and an increase in bone resorption and bone marrow fat accumulation (adipogenesis) [22]. The process of aging triggers molecular changes within BMSCs that alter their differentiation potential and impair proliferation, ultimately impairing bone regeneration and homeostasis. As individuals age, BMSCs exhibit diminished osteogenic differentiation and enhanced adipogenesis, which results in an overall reduction in bone formation [22–25]. These age-associated changes in BMSC function contribute to the onset and progression of osteoporosis, as overall bone density decreases [26]. The fate of BMSCs, including their differentiation into osteoblasts or adipocytes, is determined by an intricate balance of signaling pathways [27]. In the context of OP, this balance is disrupted, with BMSCs increasingly committing to adipogenesis rather than osteogenesis [28]. This shift in differentiation lineage is thought to exacerbate bone loss by reducing the number of osteoblasts available to form new bone. Current research suggests that the aging bone marrow microenvironment becomes increasingly adverse for BMSC function, and the over- or under-regulation of certain proteins can disrupt key signaling pathways, which further impairs osteogenesis [29]. Therefore, it is important to develop interventions to optimize the functions of BMSCs in age-related disorders, such as OP, by including approaches that aim to restore osteogenic differentiation ability [30]. In addition to the cellular fate determination, BMSCs are involved in maintaining bone homeostasis through their intercellular communication between bone elements, such as osteocytes and osteoclasts. These interactions influence bone remodeling and the regeneration processes [24,31]. The age-associated changes in BMSC function may lead to a dysregulated microenvironment that accelerates bone loss, emphasizing the need for a deeper understanding of the molecular mechanisms, proteins, and aging-related signaling pathways responsible for driving the BMSC dysfunction and differentiation into adipocytes during aging [24,25].

Although some research has been done on BMSCs in aging and osteoporosis, significant research gaps remain in our understanding of the molecular and signaling changes that occur in these cells as they age. While some studies have identified potential significant signaling pathways and expressed genes, the precise mechanisms driving the aberrations in BMSC fate and function in the aging bone marrow remain unclear. One of the key aberrations that occurs in the aged BMSC is in the BMP signaling-associated components [32]. The aged BMSCs increasingly become non-responsive to osteogenic BMP2 stimulation, while also exhibiting aberrations in the signaling pathway [32,33]. Even though the BMP receptor type 1A is upregulated in the aged BMSCs, their osteogenic potential is severely diminished [32,33]. This observation has been attributed to both the misexpression and mislocalization of the receptor on the membrane [32]. Besides, aging also promotes the activity of adipogenic transcription factors, including PPARγ, facilitating adipogenic lineage commitment in BMSC. However, the explicit mechanism of age-induced PPARγ activity is yet to be understood [34]. Additionally, senescence induced by DNA damage, telomere attrition, and increased oxidative stress, mediated by p53, has been reported to contribute to BMSC aging and may facilitate the dysfunction in cell fate and functions [34]. Again, the detailed molecular pathways that drive aging and age-associated cellular aberrations in BMSCs are still enigmatic. Additionally, the signaling networks that are affected by aging and contribute to BMSC dysfunctions are yet to be unraveled. Moreover, how the transcription regulatory networks contribute to BMSC aging is still not fully understood. These research gaps serve as a bottleneck to understanding the molecular mechanisms of BMSC aging and in the development of targeted therapeutics to slow BMSC aging and restore osteogenic potential in the aged individuals.

Therefore, this study aims to provide a comprehensive insight into the molecular changes in BMSCs that drive aging. As a model for the study, BMSCs from C57B/L (B6) mice were used, which exhibit age-associated defects in osteogenic signaling, including the BMP2 signaling pathway and diminished osteogenic potential [32,33]. Here, the phosphoproteome and total proteome of the BMSCs isolated from younger 6-month-old and older 15-month-old mice were profiled to identify changes both in protein activity and abundance. Dysregulation in protein function and expression was mapped into the molecular pathways, key gene sets, and protein-protein interaction network to identify key signaling modules, biological processes, and pathways that contribute to aging. Finally, some of the key proteins with age-associated alteration in phosphorylation and abundance level were confirmed through fluorescent immunostaining. The findings from the study provide novel insights and further refine older mechanisms into BMSC aging. These findings shed light on molecular targets to reverse age-associated BMSC dysfunction. These molecular targets can help devise therapeutic strategies to restore osteogenic potential in aged and osteoporotic individuals.

## 2. Materials and Methods

### 2.1. Acquisition of Mice for Experiments and Ethical Approval

Six-month-old (*N* = 26) and 15-month-old (*N* = 26) female C57BL/6 (B6) mice were acquired from the Translational Research Branch within the National Institute of Aging (NIA) Division of Aging Biology (Bethesda, MD, USA). After the arrival of the mice, they were kept in the Life Sciences Research Facility of the University of Delaware (Newark, DE, USA) and were allowed to acclimate to the new environment for 7 days. All mice had access to the same food and water, and five same-aged mice were kept in each cage. All the mouse-related research conducted in this study was approved by the Institutional Animal Care and Use Committee (University of Delaware, Newark, DE, USA): AUP #1194.

The 6-month-old mice represent the healthy adult stage in humans, while the 15-month-old mice represent the older, osteoporotic stage. The 15-month-old C57BL/6 (B6) mice exhibit many properties of human bone aging, such as a substantial decline in bone volume/tissue volume (BV/TV), as in osteoporotic patients, and show a similar decline in osteogenic potential [33]. The extent of decline in bone mass and bone volume in the 15-month-old mice is as pronounced as the 20-month-old mice and shares many molecular aspects, like overexpression of BMPRIa and CK2α [33]. Therefore, 15-month-old mice were chosen as a model for studying bone aging.

### 2.2. BMSC Isolation and Culture

The 6-month-old and 15-month-old mice were euthanized using CO_2_; their femurs were harvested and stored temporarily in a 1% antibiotic/antimycotic (anti/anti; Gemini Bioproducts, West Sacramento, CA, USA) solution (prepared in 1X PBS). Next, the excess muscles were removed from the femurs, and the femoral heads were sliced off using scissors. Each femur was flushed with alpha minimum essential media (Thermo Fisher Scientific, Waltham, MA, USA supplemented with 10% Fetal Bovine Serum (FBS; Gemini Bioproducts, West Sacramento, CA, USA) and 1% antibiotic/antimycotic (anti/anti; Gemini Bioproducts, West Sacramento, CA, USA) three times to isolate bone marrow stromal cells (BMSCs). Afterward, the flushed cell suspensions were disrupted into single-cell suspensions and filtered through 70 μM cell strainers (Stellar Scientific, Baltimore, MD, USA) into 50 mL conical tubes. Next, the suspensions were centrifuged at 1500 rotations per minute (RPM) for 5 min at 4 °C to concentrate the cells. After aspirating the supernatant from the top layer, the cell pellets were resuspended with αMEM supplemented with 10% FBS and 1% antibiotic/antimycotic. The cell densities were counted, and the cells were plated at 15 million cells per T175 flask (StellarScientific). The cells were grown for 4 days. On the 4th day, the serum-containing media were replaced with serum-free alpha minimum essential media containing 1% antibiotic/antimycotic. The cells were grown in αMEM, not supplemented with serum, for 24 h. The media was aspirated, sterile 1X PBS (Thermo Fisher Scientific, Waltham, MA, USA) was added, and the cells were scraped using sterile cell scrapers. The cell suspensions were taken into 2 mL microcentrifuge tubes. The tubes were centrifuged at 12,700 RPM for 20 min. The supernatant was removed, the cell pellet was dried at ambient temperature, and was later stored at −80 degrees.

### 2.3. Proteomic and Phosphoproteomic Profiling

The frozen cell pellets (15 × 10^6^) were shipped to the IDEA National Resource for Quantitative Proteomics at the University of Arkansas for Medical Sciences. Total protein from each sample went through the process of reduction, alkylation, and purification by chloroform/methanol extraction before being enzymatically digested with sequencing-grade trypsin and LysC (Promega, Madison, WI, USA). The resulting peptides were labeled using a tandem mass tag 10-plex isobaric label reagent set (Thermo Fisher Scientific, Waltham, MA, USA). Then, the TMT-labeled peptides were combined into a single multiplex group and enriched using High-Select TiO_2_ and Fe-NTA phosphopeptide enrichment kits (Thermo Fisher Scientific, Waltham, MA, USA) following the manufacturer’s instructions. Both enriched and un-enriched fractions of TMT-labeled peptides were separated into 46 fractions on a 100 × 1.0 mm Acquity BEH C18 column (Waters, Milford, MA, USA) using an UltiMate 3000 UHPLC system (Thermo Fisher Scientific, Waltham, MA, USA) with a 50 min gradient from 99:1 to 60:40 buffer A:B ratio under basic pH conditions, then consolidated into 18 super-fractions for both the enriched and un-enriched sample sets. Buffer A contained ammonium hydroxide (10 mM), acetonitrile (0.5%), and Buffer B contained ammonium hydroxide (10 mM), acetonitrile (99.9%). Both buffers A and B were adjusted to pH 10 for offline separation.

Using an UltiMate 3000 RSLCnano system (Thermofisher), each super-fraction was further separated by reverse phase XSelect CSH C18 2.5 um resin (Waters) on an in-line 150 × 0.075 mm column. A 75 min gradient from a 98:2 to 60:40 buffer A:B ratio was used to elute the peptides. Buffer B had 0.1% formic acid and 99.9% acetonitrile, while Buffer A contained 0.1% formic acid and 0.5% acetonitrile. After ionizing the eluted peptides with electrospray (2.4 kV), they were subjected to mass spectrometric analysis using multi-notch MS3 parameters on an Orbitrap Eclipse Tribrid mass spectrometer (Thermofisher). The FTMS analyzer was used to get MS data at a resolution of 120,000 over a range of 375 to 1500 *m*/*z* in top-speed profile mode. MS/MS data were obtained using the ion trap analyzer in centroid mode and normal mass range after CID activation with a normalized collision energy of 31.0. Up to ten MS/MS precursors with a normalized collision energy of 55.0 were chosen for HCD activation using synchronous precursor selection. The FTMS analyzer was then used in profile mode to acquire MS3 reporter ion data at a resolution of 50,000 over a range of 100–500 *m*/*z*.

### 2.4. Data Preprocessing

Using MaxQuant (Max Planck Institute, Martinsried, Germany, version 2.1.4.0) with a parent ion tolerance of 3 ppm, a fragment ion tolerance of 0.5 Da, a reporter ion tolerance of 0.001 Da, trypsin/P enzyme with two missed cleavages, variable modifications including oxidation on M, Acetyl on Protein N-term, and phosphorylation on STY, and fixed modification of Carbamidomethyl on C. Proteins were identified and reporter ions were quantified by searching the UniprotKB database for the species Mus musculus (6th version of 2024, Proteome ID UP000000589). Any protein identification was only accepted if it could be established with less than 1.0% false discovery rate. Proteins that were identified only by modified peptides were removed. The Protein Prophet algorithm was used to assign protein probabilities [33]. Utilizing the unenriched lysate sample, TMT MS3 reporter ion intensity values were analyzed for changes in total protein. To identify Phospho (STY) modifications, the samples were enriched for phosphorylated peptides. By using two TMT11-plex batches, the enriched and un-enriched samples were multiplexed, one for the enriched and one for the un-enriched samples.

The MS3 reporter ion intensities were normalized using ProteiNorm after conducting data acquisition and the database search [35]. The Cyclic Loess normalization method was used for normalizing the data, and the data were log2 transformed. The normalized and log2-transformed intensity data were analyzed using proteoDA, where statistical analysis was performed using Linear Models for Microarray Data (LIMMA) with empirical Bayes (eBayes) smoothing to the standard errors [36,37]. A similar approach with a few additional steps was employed for differential abundance analysis of the phosphopeptides. The phosphosites were filtered, and only peptides with a localization probability greater than 75% were retained. Peptides with zero values were filtered out, and the intensity values were log2 transformed. Differential abundance analysis of normalized and transformed phosphoproteomic data was done based on the LIMMA algorithm using the Phosphomatics web tool [38]. Similarly, differential expression analysis for proteins was performed based on the LIMMA algorithm in ProteoDA package (version 2.0).

### 2.5. Downstream Bioinformatic Analysis

The phosphorylation abundance data were used to perform kinase substrate enrichment analysis using three different tools: Phosphomatics KSEA, ROKAI-XPLORER, and KINAID, based on PhosphositePlus, SIGNOR 2.0, and Sugiyama et al. [39–44]. Kinases predicted by the tools were filtered based on their differential protein expression and phosphorylation between the age groups. Transcription factor enrichment analysis was performed using the Molecular Signature Database’s Gene Set Enrichment Analysis tool and the iRegulon Tool in Cytoscape 3.0 (default parameters) to identify the transcription factors acting upstream of the differentially expressed proteins [45–50]. Among the predicted transcription factors, only those significant in differential analysis and supervised clustering were selected. Proteins with significantly differential phosphorylation and/or expression based on both differential abundance analysis and supervised clustering were used for functional pathway enrichment analysis using the ShinyGO 0.85.1 web tool based on KEGG, Reactome, WikiPathways, and Gene Ontology databases [51–55]. The same set of proteins was also used to reconstruct the protein-protein interaction network based on the STRING v12.0 database (excluding text-mining-based interactions) [56]. The network was imported into Cytoscape 3.0, where the MCODE tool (default parameters) was used to identify functionally important protein modules [57]. Each module was annotated for its function using Gene Ontology: Biological Process terms.

### 2.6. Fluorescent Immunostaining

Frozen BMSCs were thawed by suspending in warm αMEM (containing 10% FBS and 1% antimycotic/antibiotic solution) and were centrifuged for 5 min at 1500 RPM. The cell pellet was resuspended in αMEM, and the cells were grown in the six-well plate over 22 mm round coverslips. The cells were grown for 5 days without any media change. Then, the media was removed by aspiration, and the cells were rinsed with ice-cold 1X PBS. Cell fixation was done with 4% PFA (pH 7) at room temperature for 15 min. Next, the BMSCs were permeabilized using 0.1% saponin solution (in sterile DI water) for 20 min at ambient temperature. Afterwards, BMSCs were incubated with 3% BSA/ 0.1% Saponin (in 1X PBS) on ice for around 1 h to prevent non-specific antibody binding. Next, the cells were incubated with the primary, mouse anti p-Erk1/2 antibody (Santa Cruz Biotechnology (Dallas, TX, USA), sc-7383) at a 1:100 dilution (in 1% BSA/0.1% saponin) for 1 h 15 min at room temperature. Additionally, a coverslip with BMSC was designated as the “secondary control,” and was also incubated with 1% BSA/0.1% saponin without any primary antibodies. The samples (including the secondary control sample) were incubated with the secondary antibody Alexa Fluor 488 conjugated donkey anti-mouse (Molecular Probes (Eugene, OR, USA), Cat# A21202) at a 1:1000 dilution (in 1% BSA/0.1% saponin) for 1 h at room temperature, following the incubation in anti p-Erk1/2 antibody or 1% BSA/0.1% saponin. The samples were rinsed with 1X PBS after the incubation. Afterward, nuclear staining was performed by incubating the cells with Hoechst 3342 (Fisher Scientific (Hampton, NH, USA, Cat# H1399) at a 1:5000 dilution (in deionized water) for 10 min. After three times 1X PBS wash, Airvol mounting media was used to mount the coverslips on the glass slides. The mounting media was allowed to harden for over 2 days in a dark area. Finally, using the 63X oil immersion objective lens of a Zeiss LSM880 Confocal Microscope (Zeiss, Oberkochen, Germany), images were taken at different areas within the slides.

For preparing bone sections for fluorescent immunohistochemistry, the entire femurtibia was harvested from the mice after they were euthanized with CO_2_. The bones were fixed in 10% Neutral Buffered Formalin (NBF) (Epredia, Kalamazoo, MI, USA, Cat #5701) for 24 h. The bones were rinsed with 1X PBS and decalcified for 5 days in a 10% EDTA (*w*/*v*) solution. The EDTA solution was changed every 2 days. Following decalcification, the decalcified bone samples were stored in 70% ethanol and sent to the Sam and Ann Barshop Institute for Longevity and Aging Studies at San Antonio, Texas. The bone samples were cleared there and were embedded with paraffin. Afterwards, the formalin-fixed paraffin-embedded (FFPE) mouse bone specimens were sectioned at 5 μm thickness. Sectioned samples were warmed in a heating block at 100 degrees Celsius for 2 min. Later, the sections were cooled down at room temperature. The sections were deparaffinized and rehydrated by incubating in 100% xylene in two rounds, each for 15 min, and then in a series of 100% ethanol, 96% ethanol, and 70% ethanol for 7 min each. Finally, the sections were kept in deionized water for 5 min. The sections were incubated in warm testicular hyaluronidase solution (MilliporeSigma, Burlington, MA, USA, Cat# H3884–100MG) at 37 °C for approximately 40 min to retrieve antigens. Then, the sections were incubated in 3% BSA solution containing 0.1% Saponin for 1 h at ambient temperature in order to block nonspecific antibody binding. To label Erk1/2 fluorescently, the bone sections were incubated with the primary mouse anti-Erk1/2 antibody (St John’s Laboratory Ltd. (London, UK), STJ97783) at a 1:100 dilution (in 1% BSA/0.1% saponin) for 1 h and 25 min at room temperature. Additionally, sections designated as the “secondary control” were also incubated in 1% BSA/0.1% saponin without any primary antibody. After incubation in anti-Erk1/2 antibody or 1% BSA/0.1% saponin, the section samples (including the secondary control samples) were rinsed twice in 1X PBS and were incubated with the secondary antibody CoraLite 488 goat anti-mouse (Protein Tech (Rosemont, IL, USA), SA00013–1) at a 1:500 dilution (in 1% BSA/0.1% saponin) at ambient temperature for approximately an hour. Next, nuclear staining was performed with Hoechst 3342 (Fisher Scientific (Hampton, NH, USA), Cat# H1399) at a 1:5000 dilution (in deionized water) for approximately 10 min at room temperature. The excess staining solution was removed by rinsing the sections thrice in 1X PBS. Airvol mounting media was used to mount the coverslips on the glass slides. The mounting media was allowed to harden for over 2 days in a dark area. Finally, using the 63× oil immersion objective lens of a Zeiss LSM880 Confocal Microscope (Zeiss, Oberkochen, Germany), images were taken at different areas within the slides. The images of both immunostained cells and bone sections were analyzed using ZEISS ZEN Lite software (version 3.1).

### 2.7. Statistical Analyses

The phosphoproteomic and total proteomic intensity data were normalized using the cyclic loess method and were log2 transformed. Statistical analysis was performed using Linear Models for Microarray Data (LIMMA) with empirical Bayes (eBayes) smoothing for the standard errors. Raw *p*-values were corrected for multiple hypothesis testing using the Benjamini–Hochberg method, and adjusted *p*-values less than 0.05 were considered as a measure of significance for differential abundance analysis. Partial Least Squares-Discriminant Analysis (PLS-DA) was performed on significantly differentially abundant proteins/phosphoproteins. Variable Importance in Projection (VIP) score for each protein or phosphoprotein was calculated across the most important components (Components 1 and 2). For defining proteins and phosphoproteins significantly altered with aging, a dual filter strategy was used: VIP score > 1.0 and BH-adjusted *p*-value < 0.05. This two-step filtering strategy was used to identify features that were both statistically significant and important for group discrimination, and these features were subsequently used for functional enrichment and network analyses. For functional enrichment, the *p*-values were also corrected using the Benjamini–Hochberg method. For most kinase–substrate enrichment analyses, statistical significance was evaluated using raw *p*-values rather than multiple testing–adjusted values. This approach was adopted due to the limited coverage and ongoing development of kinase–substrate annotation databases, which may reduce statistical power after correction and increase the likelihood of false negatives.

## 3. Results

### 3.1. Aging Reprograms the Phosphoproteome of BMSC

**Figure 1.**
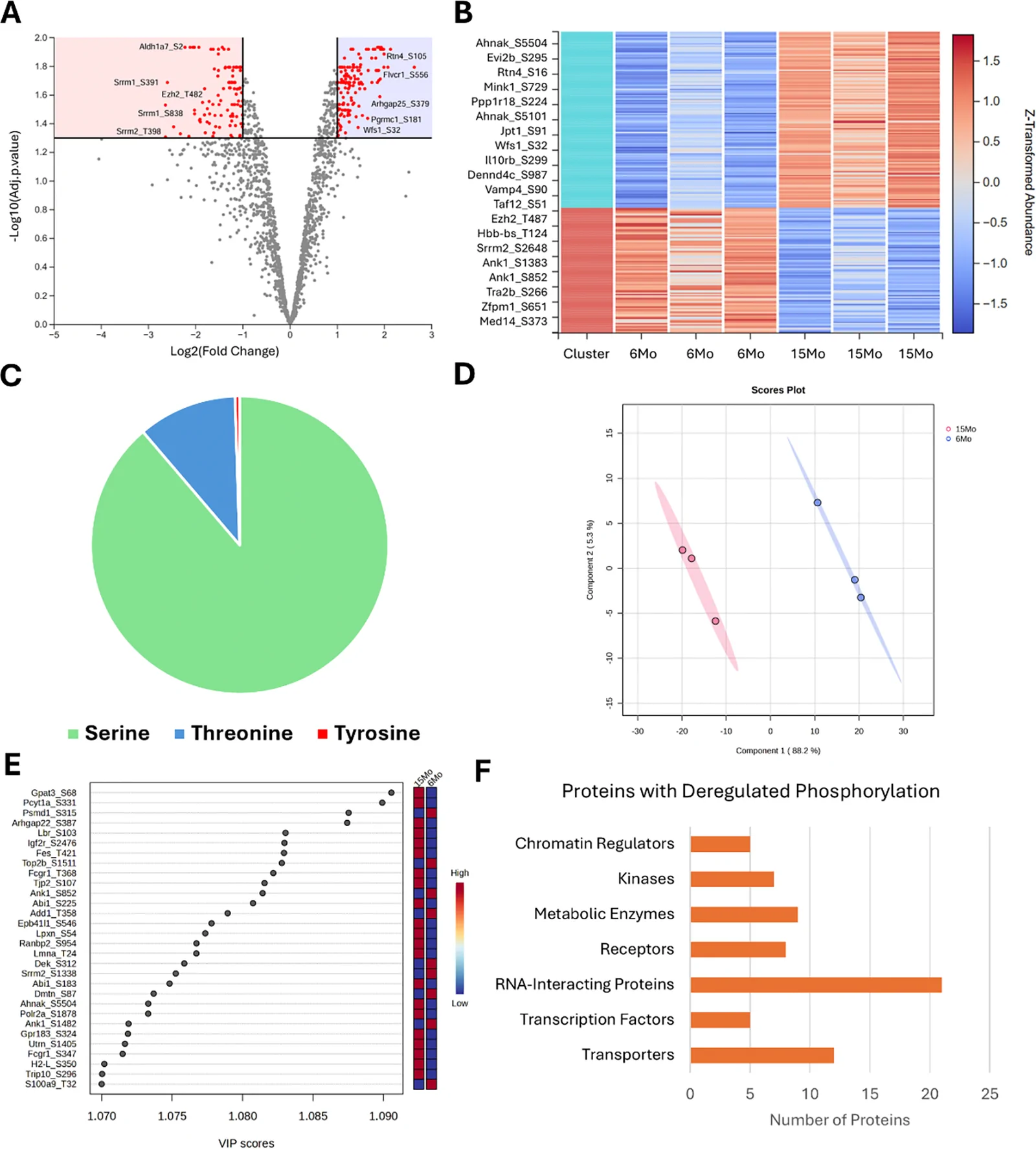
Phosphoproteomic profiling of 6-month-old and 15-month-old murine BMSC. (**A**) Volcano plot depicting the differentially phosphorylated phosphosites between aged and young BMSC (Red box = downregulation; Purple box = upregulation; BH-adjusted *p*-value < 0.05). (**B**) Heatmap of phosphosites that shows a distinct age-specific phosphorylation pattern in BMSC. Phosphosites belonging to the teal cluster show increased phosphorylation with age, while phosphoproteins in the red cluster show reductions in phosphorylation. (**C**) Relative abundance of three major phosphosites that underwent deregulated phosphorylation with aging. (**D**) 2D Score plot for the two age groups after partial least squares-discriminant analysis, showing distinct clustering of samples. (**E**) VIP (Variable Importance in Projection) scores for the phosphosites, illustrating their contribution to the age-specific phosphoproteomic profiles. (**F**) Categorization of significantly deregulated phosphoproteins (BH-adjusted *p*-value < 0.05 and PLS-DA VIP Score > 1.0) into major functional classes.

The phosphoproteomic profiling of bone marrow mesenchymal stem cells (BMSC), combined with downstream statistical filtering, resulted in the mapping of 1792 unique phosphosites. Principal component analysis reveals distinct clustering of the replicates for both 6-month-old BMSC and 15-month-old BMSC, indicating good data quality and reproducibility (Figure S1). A majority of the identified phosphosites were serine/threonine phosphosites, followed by tyrosine phosphosites (Figure S1). Differential abundance analysis on the cyclic loess-normalized and log2-transformed intensities of phosphosites between 15-month-old and 6-month-old BMSC revealed a total of 400 phosphosites that were significantly deregulated (BH-adjusted *p* value < 0.05) (Figure 1A). On 188 unique proteins, 243 unique phosphosites had elevated phosphorylation, while 157 unique phosphosites on 119 unique proteins had reduced phosphorylation with aging. The volcano plot in Figure 1A shows some of the most significant changes in the phosphorylation pattern between aged and young BMSC. Interestingly, all the unregulated phosphorylations were seen in serine/threonine phosphosites, while 155 serine/threonine phosphosites, along with two tyrosine phosphosites, had downregulation in phosphorylation (Figure S1). Proteins with upregulated phosphorylation include Reticulon-4 (Rtn4_S105), Choline/ethanolamine transporter FLVCR1 (Flvcr1_S556), Rho GTPase-activating protein 25 (Arhgap25_S379), Membrane-associated progesterone receptor component 1 (Pgrmc1_S181), and Wolframin (Wfs1_S32). In contrast, Aldehyde dehydrogenase, cytosolic 1 (Aldh1a7_S2), Serine/arginine repetitive matrix protein 1 (Srrm1_S391), Histonelysine N-methyltransferase EZH2 (Ezh2_T482), and Serine/arginine repetitive matrix protein 2 (Srrm2_T398) are some of the proteins whose phosphorylation was significantly downregulated with aging. Some of the key phosphosites show a distinct phosphorylation pattern across replicates in the two age groups (Figure 1B). Alteration in phosphorylation due to aging was most pronounced in serine phosphosites (accounting for 88% of the changes), followed by threonine (11%) and tyrosine (1%), respectively.

Supervised clustering based on the Partial Least Squares Discriminant Analysis (PLS-DA) method was done on the phosphoproteins with significantly altered abundance levels to identify the age-correlated phosphoproteomic signatures in BMSC. A total of 213 phosphosites were identified to be key signatures that show age-correlated phosphorylation (VIP score > 1.0). Annotations for the biological activities of the key feature proteins revealed that the most abundant protein group with altered phosphorylation is RNA-binding proteins, followed by transporter proteins and metabolic enzymes, respectively (Figure 1F). Some of the most important phosphosites that exhibit age-specific increase in phosphorylation are Gpat3_S68 (Glycerol-3-phosphate acyltransferase 3), Pcyt1a_S331 (Choline-phosphate cytidylyltransferase A), and Arhgap22_S387 (Rho GTPase-activating protein 22) (Figure 1E). Similarly, the top three most significant phosphoproteomic signatures that correspond to age-associated changes and decrease in abundance are Psmd1_S331 (26S proteasome non-ATPase regulatory subunit 1), Top2b_S1511 (DNA topoisomerase 2-beta), and Ank1_S852 (Ankyrin-1) (Figure 1E).

### 3.2. The Activity of Key Kinases Is Dysregulated in Aged BMSC

**Figure 2.**
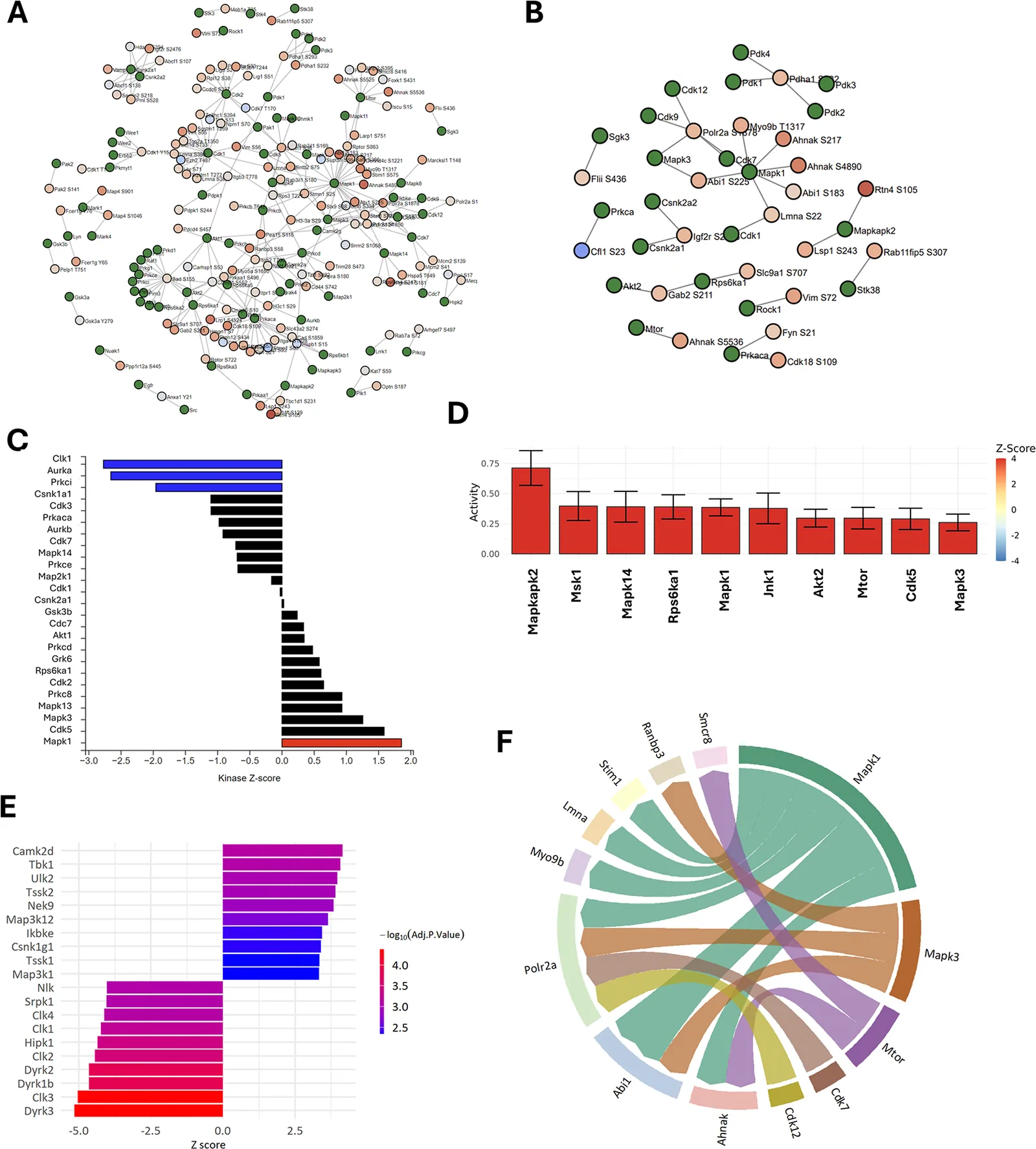
Upstream kinases mediating alterations in phosphorylation dynamics in aged BMSC. (**A**) Kinase-substrate interaction network showing enriched kinases and their substrates with alterations in phosphorylation (Red = Upregulation in phosphorylation with age; Blue = Downregulation in phosphorylation with age; Green = Upstream kinases). (**B**) A closer view of the kinase-substrate network, which only includes significantly deregulated phosphoproteins (BH-adjusted *p*-value < 0.05 and PLS-DA VIP Score > 1.0) and their upstream kinases. (**C**) Predicted upstream kinases and their estimated relative activity in the aged BMSCs based on KSEA (Red = significantly higher activity; Blue = significantly lower activity; Black = insignificant). (**D**) ROKAI-XPLORER predicted upstream kinases, their relative activity, and Z-score. (**E**) Orthology-based prediction of kinases mediated the dysregulation in phosphorylation dynamics in the aged BMSCs. (**F**) Upstream kinases with significant alteration in phosphorylation and/or expression levels (Raw *p*-value < 0.05) and their substrates that underwent significant alteration in phosphorylation (BH-adjusted *p*-value < 0.05) (Dark Color = Kinases; Light Color = Substrates).

As an age-associated change in the phosphorylation pattern of proteins was found, it was hypothesized that the upstream kinases acting on those proteins might have altered activity with aging as well. To quantify the change in kinase activity, the kinase substrate enrichment was performed, and the kinase-substrate interaction network was reconstructed. The full kinase-substrate network is depicted in Figure 2A, while Figure 2B illustrates the network of substrates identified to be significant after supervised clustering analysis and their upstream kinases. The most connected kinase in the network, with 22 phosphosite substrates on 18 different proteins, is Mapk1 (Mitogen-activated protein kinase 1/Extracellular signal-regulated kinase 2/ERK2) (Figure 2A). Some of its substrates, which underwent differential phosphorylation due to aging, include Ahnak (AHNAK nucleoprotein), Polr2a (DNA-directed RNA polymerase II subunit RPB1), Stat1 (Signal transducer and activator of transcription 1), Rptor (Regulatory-associated protein of mTOR), and Bcl6 (Ribosome biogenesis protein SLX9 homolog). In addition, the network also includes kinases like Cdk1 (Cyclin-dependent kinase 1), Cdk2(Cyclin-dependent kinase 1), Mtor (Mechanistic target of rapamycin), Prkaca (cAMP-dependent protein kinase catalytic subunit alpha), Prkca (Protein kinase C alpha type), and Mapk3 (Mitogen-activated protein kinase 3/Extracellular signal-regulated kinase 1/ERK1). Kinase-substrate enrichment analysis was performed based on the normalized phosphoproteomic data using the KSEA tool of the Phosphomatics webserver, ROKAI-XPLORER web tool, and KINAID to identify upstream kinases that mediate alterations in phosphorylation with age. KSEA predicted that three kinases, namely Clk1 (CDC-like kinase 1), Aurka (Aurora Kinase A), and Prkci (Protein kinase C, iota), would have decreased activity with aging, while Mapk1 was predicted to have higher activity in aged BMSC (Figure 2C). Similarly, the ROKAI-XPLORER tool predicted a total of 10 kinases having enhanced activity in aged BMSC compared to younger ones. These kinases include Mapk1, Mapk3, Mtor, Mapkapk2 (MAP kinase-activated protein kinase 2), and Mapk14 (Mitogen-activated protein kinase 14) (Figure 2D). The KINAID tool, which uses peptide sequence and orthology-based searching, predicted a total of 84 kinases to have significantly altered activity (adjusted *p*-value < 0.05) due to aging in BMSC. Among them, 50 were predicted to have an increase in substrate phosphorylation activity, while 34 had a decrease in activity. The top altered kinases with an age-associated spike in activity include Map3k1 (Mitogen-activated protein kinase kinase kinase 1), Map3k12 (Mitogen-activated protein kinase kinase kinase 12), and Camk2d (Calcium/calmodulin-dependent protein kinase II, delta), whereas some of the most significant kinases with downregulated activity are Clk1 (Cdc-like kinase 1), Dyrk3 (Dual-specificity tyrosine phosphorylation regulated kinase 3), and Srpk1 (Serine/arginine-rich protein specific kinase 1) (Figure 2E).

Among the kinases predicted to have altered activity due to aging based on KSEA, ROKAI-XPLORER, and KINAID, a total of four were predicted by two different tools. There was no kinase inferred by all three tools. The four kinases were Mapk1 (inferred by KSEA and ROKAI-XPLORER), Clk1 (inferred by KSEA and KINAID), Cdk5, and Mapkapk2. Both KINAID and ROKAI-XPLORER predicted that Cdk5 and mapkapk2 undergo significant alteration in their activity with aging based on phosphoproteomics data.

Among the kinases predicted to have differential activity between the age groups, five were found to have significant (*p* value < 0.05) alteration in phosphorylation and/or expression levels based on phosphoproteome and whole proteome profiling (Figure 2F). The kinases are Mapk1, Mapk3, Mtor, Cdk7 (Cyclin-dependent kinase 7), and Cdk12 (Cyclin-dependent kinase 12). These kinases together had a total of eight substrate proteins that underwent deregulated phosphorylation due to aging (Adjusted *p* value < 0.05). The most common substrate is Polr2a, while some others are Ahnak, Abi1 (Abl interactor 1), Stim1 (Stromal interaction molecule 1), and Lmna (Lamin A) (Figure 2F).

### 3.3. Aged BMSCs Exhibit a System-Wide Change in Protein Expression

**Figure 3.**
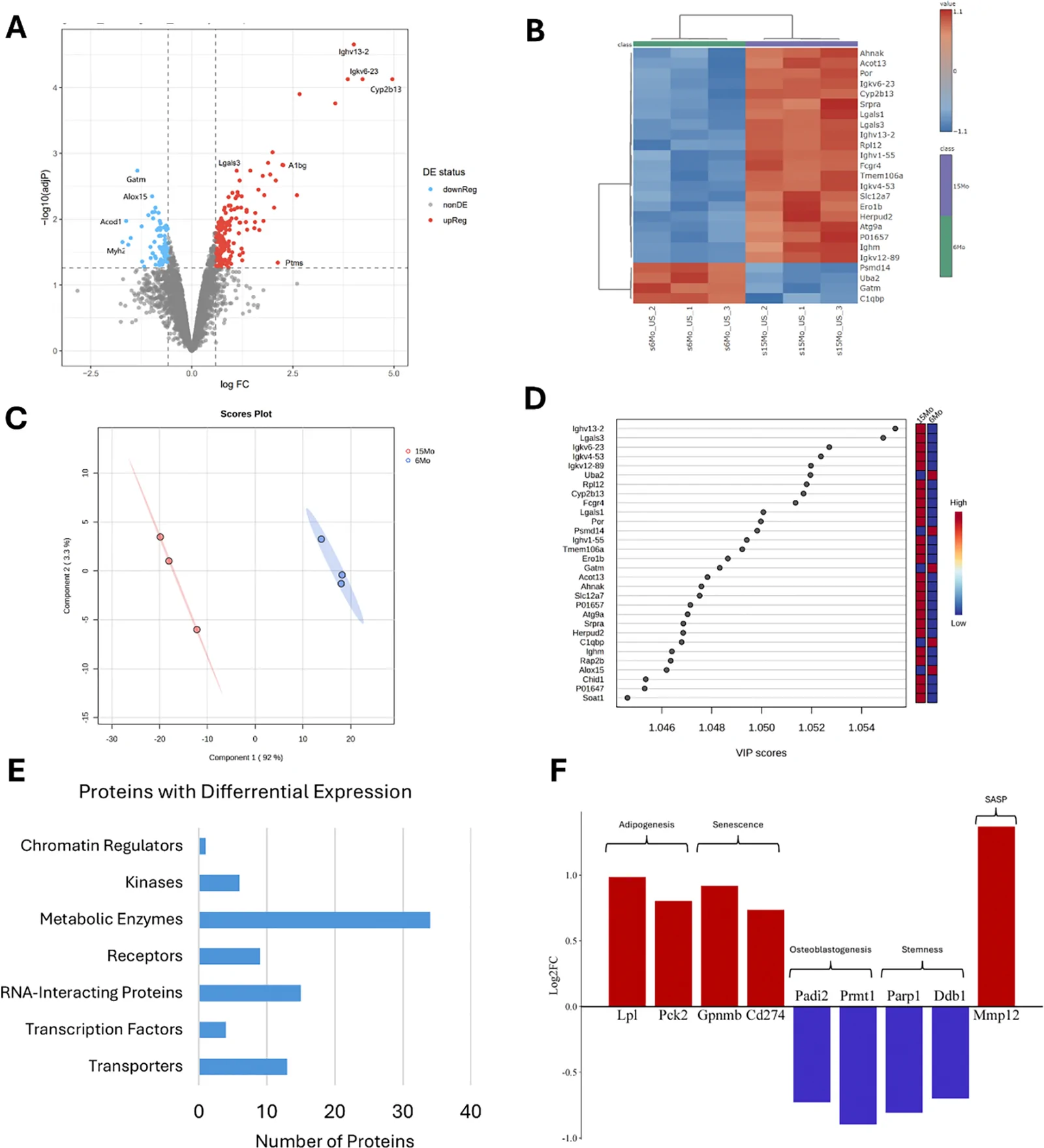
Expression analysis of the total proteome of BMSCs shows age-associated alterations. (**A**) Differential protein expression analysis between aged and young murine BMSC depicted in a volcano plot, where upregulated proteins are in red while downregulated proteins are in blue (BH-adjusted *p*-value < 0.05). (**B**) Top 25 most significantly mis-expressed proteins between the aged and young BMSC based on adjusted *p*-value. (**C**) Partial-least-square discriminant analysis on the proteomic expression data reveals distinct age-specific protein expression. (**D**) Top 25 significantly mis-expressed proteins (BH-adjusted *p*-value < 0.05) based on VIP scores (VIP Score > 1.0). (**E**) Classification of significantly differentially expressed proteins (BH-adjusted *p*-value < 0.05 and VIP score > 1.0) based on major biological functions. (**F**) Expression profiles of markers for osteogenic differentiation, adipogenesis, senescence, stemness, and senescence-associated secretory phenotype (SASP) (Red = Upregulation; Blue = Downregulation).

Profiling of the proteome from both aged and young BMSC led to the identification of 4064 statistically significant and unique proteins after mapping against the mouse proteome and statistical filtering. Principal component analysis was performed on the cyclic-loess normalized log2-transformed protein intensity data, which revealed individual clustering of the replicates for each age group (6-month-old BMSC and 15-month-old). This indicates good data quality and reproducibility (Figure S3). Differential protein expression analysis between the two age groups identified a total of 374 dysregulated proteins, among which 256 proteins were upregulated while 122 were downregulated in the aged BMSCs compared to the young BMSCs (BH adjusted *p*-value < 0.05) (Figure 3A). Some of the most significant overexpressed proteins include Ighv13–2 (Immunoglobulin heavy variable 13–2), Cyb2b13 (Cytochrome P450, family 2, subfamily b, polypeptide 13), A1bg (Alpha-1-B glycoprotein), and Ptms (Parathymosin), whereas Gatm (glycine amidinotransferase/L-arginine: glycine amidinotransferase), Alox15 (Arachidonate 15-lipoxygenase), Acod1 (Aconitate decarboxylase 1), and Myh2 (Myosin-2, heavy chain) are some of the most highly downregulated proteins in aged BMSC. The heatmap in Figure 3B shows the top 25 most significantly mis-expressed proteins due to aging in BMSCs. These proteins show similar patterns in terms of alteration across all replicates within the age groups (Figure 3B). Some of these proteins with distinct age-specific expression patterns are Ero1b (Endoplasmic Reticulum oxidoreductase 1 beta), Psmd14 (26S proteasome non-ATPase regulatory subunit 14), Atg9a (Autophagy related 9a), Uba2 (Ubiquitin-like modifier activating enzyme 2), and Slc12a7 (Solute carrier family 12, member 7), which are involved in diverse biological processes within the cell.

Supervised clustering analysis based on the Partial Least Squares Discriminant Analysis (PLS-DA) method was performed on the protein intensity matrix containing only significantly dysregulated proteins to identify the age-correlated protein signatures in BMSC. The clustering analysis revealed distinct clusters for the samples within the two age groups, with the principal components 1 and 2 combinedly corresponding to more than 95% of the variation between the two biological age groups in the filtered data (Figure 3C). The PLS-DA clustering also calculated the Variable Importance in Projection (VIP) scores for all the proteins, denoting the relative contribution of proteins to group separation. Among the 374 proteins that were dysregulated with aging, a total of 206 proteins were identified to be significant (VIP score > 1.0) in their contribution to the age-specific proteomic signatures. Some of the upregulated proteins with the highest contribution to BMSC aging and associated proteomic signatures are Ighv13–2, Lgals3 (Lectin, galactose binding, soluble 3), Atg9a, and Slc12a7, while some of the downregulated proteins with significant contribution to age-specific proteome changes are Uba2, Alox15, and Psmd14 (Figure 3D).

When the 206 significantly dysregulated proteins based on PLS-DA were annotated for their biological functions, a majority (34 proteins) were identified to be involved in cellular metabolic processes (Figure 3E). Some of these proteins are Alox15, Acod1, Gatm, Pdhb (Pyruvate dehydrogenase E1 component subunit beta, mitochondrial), Acads (Acyl-Coenzyme A dehydrogenase, short chain), and Lpl (Lipoprotein lipase). The second and third most represented protein classes were RNA-binding proteins and transporter proteins, respectively (Figure 3E). Proteins within these classes included Slc12a7, Clcn7 (H(+)/Cl(−) exchange transporter 7), Atp1a3 (ATPase, Na^+^/K^+^ transporting, alpha 3 polypeptide), as well as G3bp (G3BP stress granule assembly factor 1), Cirbp (Cold-inducible RNA-binding protein), and Slirp (SRA stem-loop-interacting RNA-binding protein, mitochondrial). In addition, aged BMSCs also exhibit a statistically significant elevation in the expression of key adipogenesis genes like Lpl and Pck2 (Phosphoenolpyruvate carboxykinase 2, mitochondrial), as well as markers for cellular senescence, including Gpnmb (Glycoprotein (transmembrane) nmb) and Cd274, and senescence-associated secretory phenotype (SASP) like Mmp12 (Matrix metalloprotease 12) (Figure 3F). Simultaneously, a decrease in the expression of genes involved in osteoblastogenic differentiation, like Padi2 (Peptidyl arginine deiminase, type II), Prmt1 (Protein arginine N-methyltransferase 1), and stemness maintenance, like Parp1 (Poly [ADP-ribose] polymerase 1), and Ddb1 (DNA damage-binding protein 1) were also observed with aging in BMSC (Figure 3F).

### 3.4. Transcription Factor Activity and Expression Are Dysregulated with Aging

**Figure 4.**
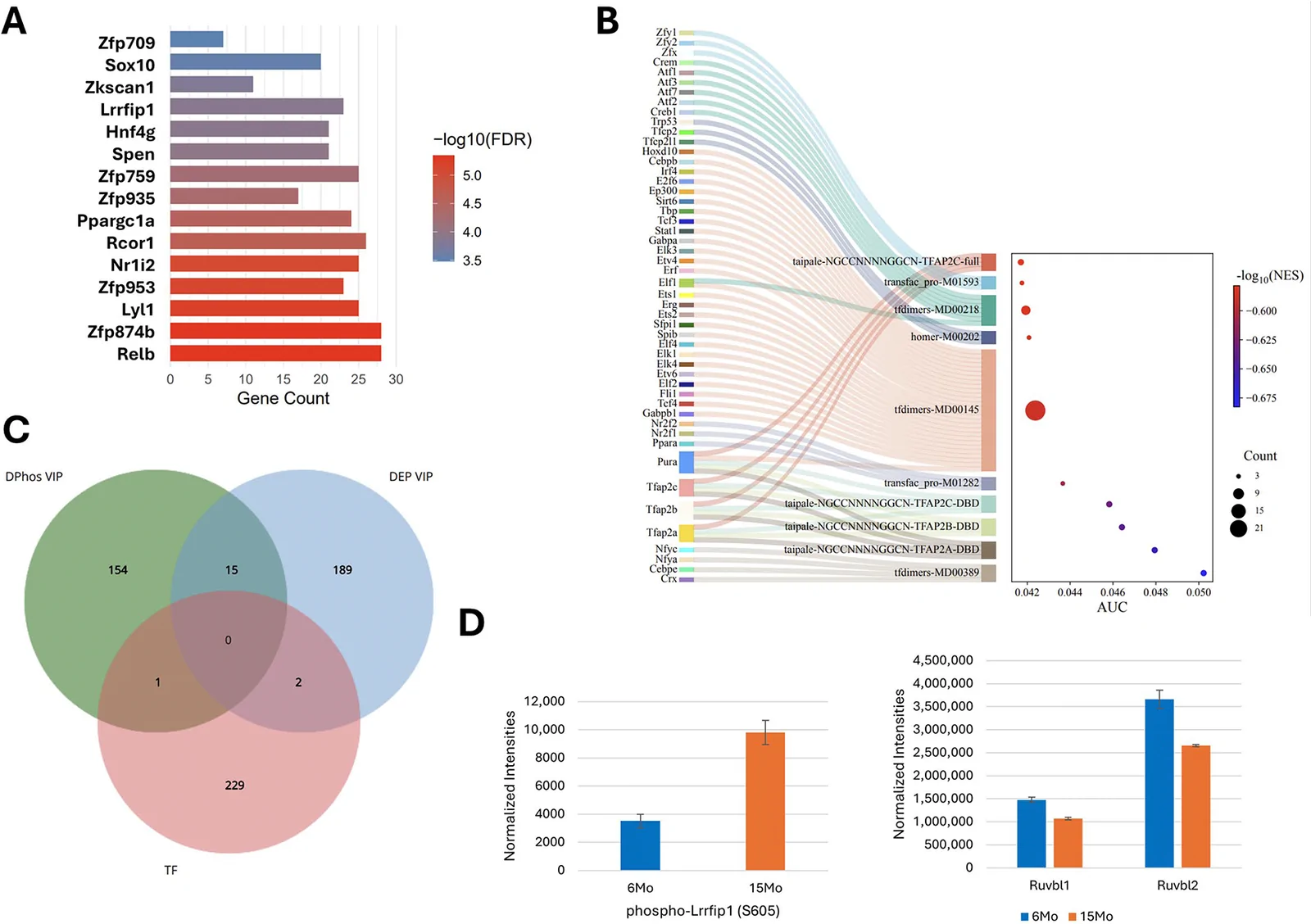
Dysregulation in transcription factor activity in BMSC due to aging. (**A**) Enriched transcription factors in the upstream of significantly differentially expressed proteins (BH-adjusted *p*-value < 0.05 and VIP score > 1.0) based on gene set enrichment analysis. (**B**) Enriched sequence motifs and motif-binding transcription regulators in the regulatory elements of differentially expressed proteins. (**C**) Transcription factors with age-associated deregulation in activity (phosphorylation) and expression. (**D**) Phosphorylation and abundance level of the deregulated transcription factors (BH-adjusted value < 0.05).

To identify the transcription factors that mediate the age-associated reprogramming of the BMSC proteome, gene set enrichment analysis (GSEA) was performed using the proteins that were significantly differentially expressed between the old and young BMSC. The GSEA analysis identified a total of 90 significantly enriched (False Discovery Rate, FDR < 0.05) transcription factors that act on the proteins that were substantially dysregulated (BH adjusted *p*-value < 0.05 and VIP Score > 1.0) with aging in BMSC. The top 15 most significant transcription factors have been illustrated in Figure 4A, which includes zinc finger proteins like Zfp709, Zfp759, and Zfp874B. In addition, other classes of transcription factors were also identified to be significantly regulating the mis-expressed proteins, such as Lrrfip1 (Leucine rich repeat (in FLII) interacting protein 1), Sox10 (SRY (sex determining region Y)-box 10), Hnf4g (Hepatocyte nuclear factor 4, gamma), Ppargc1a (Peroxisome proliferative activated receptor, gamma, coactivator 1 alpha), and Rcor1 (REST corepressor 1). A different gene-centric approach was also employed to identify transcription regulatory proteins that bind to the regions up to 20 kilobases upstream and downstream of the transcription start sites of the dysregulated protein-encoding genes. The transcription factor enrichment analysis identified a total of 77 enriched transcription factor-binding motifs to significant in the regulatory elements of the genes with altered expression. The top 10 significantly enriched sequence motifs (FDR < 0.001) have been illustrated in Figure 4B along with their corresponding transcription regulators. Some of the transcription factors that bind the enriched motifs and are predicted to be the mediator of age-associated alterations in gene expression are Pura (Purine rich element binding protein A), Tfap2a (Transcription factor AP-2, alpha), Tfap2c (Transcription factor AP-2, gamma), Cebpe (CCAAT/enhancer binding protein epsilon), and Ppara (Peroxisome proliferator activated receptor alpha) (Figure 4B). A total of 155 transcription regulatory proteins were predicted to bind the enriched sequence motifs. To identify the transcription factors among the predicted ones that show age-associated alteration in activity, their abundance and phosphorylation patterns were analyzed using the proteome and phosphoproteome level data. Three transcription factors were identified to have either dysregulation in phosphorylation or expression with aging (Figure 4C).

Lrrfip1 was the only transcription factor that underwent differential phosphorylation in aged BMSC, where the phosphorylation level increased more than two-fold compared to the young BMSC (Figure 4D). In contrast, the expression of the transcription factors Ruvbl1 and Ruvbl2 was downregulated due to aging in BMSC (Figure 4D). Together, these three transcription factors regulate the expression of 42 mis-expressed proteins (Figure S2). Lrrfip1 regulates a total of 24 proteins’ expression, among which 10 underwent downregulation, and the rest had an upregulation (Figure S2). Similarly, among the 12 proteins whose expression is regulated by Ruvbl1, three were downregulated while nine were upregulated (Figure S2). A total of 14 Ruvbl2-regulated proteins showed overexpression with aging, while four saw a decrease in expression levels (Figure S2).

### 3.5. Aging Alters the Activity of Key Biological Processes and Pathways in BMSC

**Figure 5.**
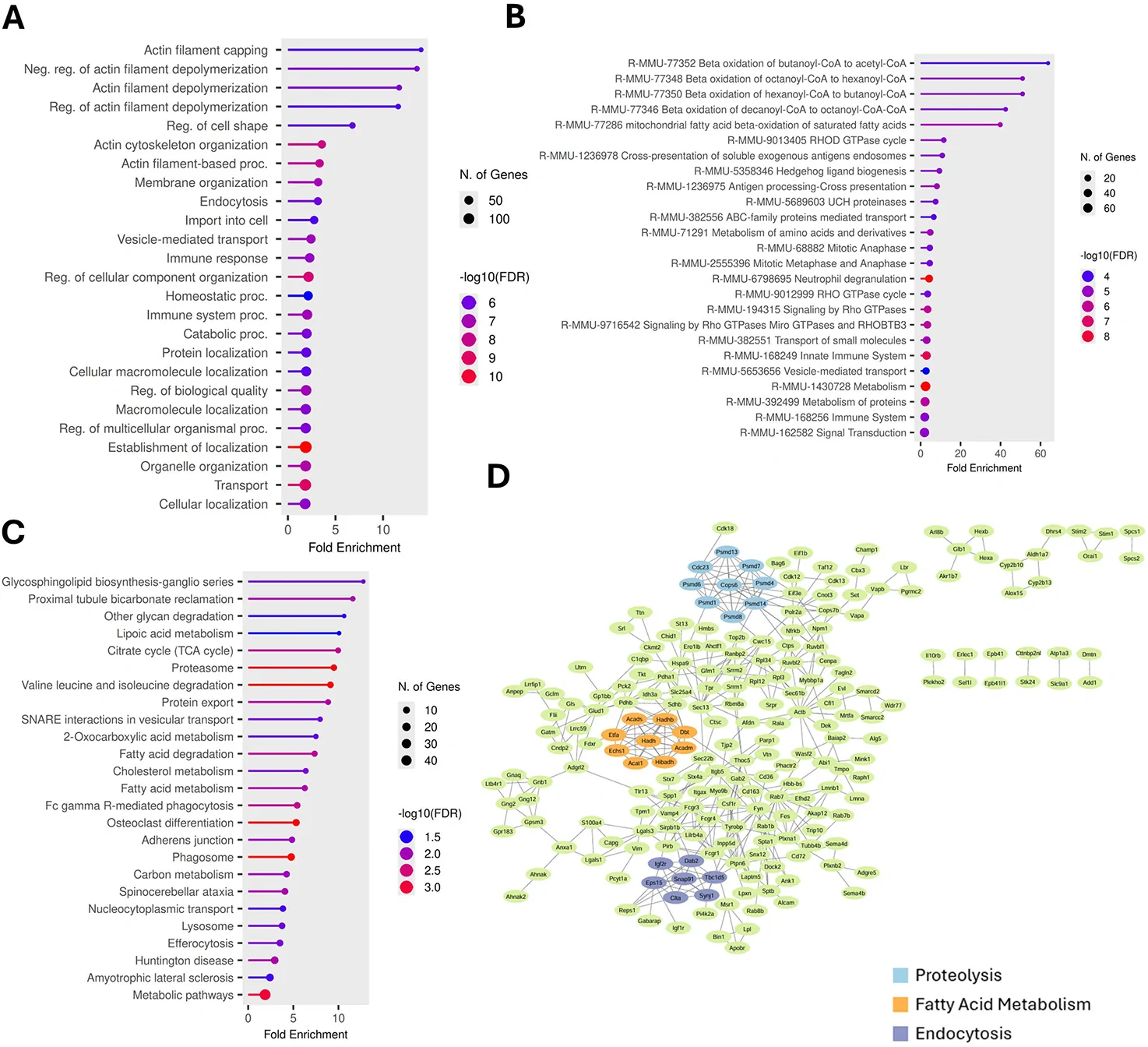
Aging affects numerous biological processes and intracellular pathways in BMSCs. Functional enrichment analysis was performed based on Gene Ontology: Biological Process annotations (**A**), KEGG Mouse Pathway Database (**B**), and Reactome Database (**C**). (**D**) Top three functional protein–protein interaction modules/cluster and their biological functions. (False Discovery Rate < 0.05).

Cellular pathways and biological processes affected by aging in BMSC were identified through over-representation analysis and network-biology-based approaches. The Gene Ontology: Biological Process-based enrichment analysis revealed that processes associated with actin filament regulations, such as actin filament capping, depolymerization, and organization is impacted in aged BMSCs, implying a change in cellular motility, intracellular transport mechanisms, and other cell properties (Figure 5A). Interestingly, an associated process that was affected by aging was vesicular transport, including Endocytosis, Vesicle-mediated transport, and Transport (Figure 5A). Cellular processes that regulate the localization of macromolecules, including proteins, were also predicted to be deregulated with aging in BMSC (Figure 5A).

KEGG pathway database for the mouse predicted that an age-associated metabolic reprogramming occurs in BMSCs. Some of the key metabolic pathways that were affected include Glycosphingolipid biosynthesis-ganglio series, Glycan degradation, Lipoic acid metabolism, Citrate cycle, Fatty acid metabolism, Cholesterol metabolism, and Glycosaminoglycan degradation (Figure 5B). Another pathway that was pronounced here was the SNARE interaction in vesicular transport, further implicating vesicular transport mechanisms as one of the key dysregulated biological processes. Another transport mechanism that was altered due to aging was the nucleocytoplasmic transport across the nuclear envelope (Figure 5B). To identify a more specific pathway module, the Reactome pathway database was used. The top 25 most significant pathways (FDR < 0.05) have been depicted in Figure 5C. The most significantly enriched pathways are involved in fatty acid metabolism, including β-oxidation of butanoyl-CoA to acetyl-CoA and earlier stages of the process (Figure 5C). In addition, amino acid metabolism, small molecule transport, and ABC transporter-mediated transport mechanisms were also affected by aging (Figure 5C). Among the signaling cascades that were impacted in aged BMSCs, RHO GTPase Signaling, Hedgehog ligand-mediated signaling, Dectin-1 mediated noncanonical NF-kB signaling, and MAPK4/MAPK6 signaling are the most prominent (Figure 5C). Like the other two pathways databases, the Reactome database also predicted a significant dysregulation in endocytic and vesicular transport pathways (Figure 5C). Another important set of processes that changed due to aging are the mitotic cell division-associated processes, including FBXL7 down-regulating AURKA during mitotic entry and in early mitosis, p53-Dependent G1/S DNA damage checkpoint, and the role of GTSE1 in G2/M progression after the G2 checkpoint (Figure 5C). In addition, several transcription regulatory mechanisms were also found to be dysregulated in aged BMSC, such as Regulation of RUNX2 expression and activity, Transcriptional reg. by RUNX2, and Transcriptional reg. by RUNX3. Misregulation of proteasomal degradation was another key process seen in aged BMSC according to KEGG and Reactome databases (Figure 5B,C). To complement these findings, a network-topology-based process was also employed, which identified highly interconnected functional protein interaction modules. Among the modules, the top three highest-ranked modules were annotated for their biological functions based on Gene Ontology: Biological Process terms, and the modules were primarily involved in three distinct functions: Proteolysis, Fatty Acid Metabolism, and Endocytosis (Figure 5D). In addition, a large, highly connected protein module was found to mediate nucleocytoplasmic transport of molecules like RNAs and proteins.

### 3.6. ERK1/2 Activity Increases with Aging

**Figure 6.**
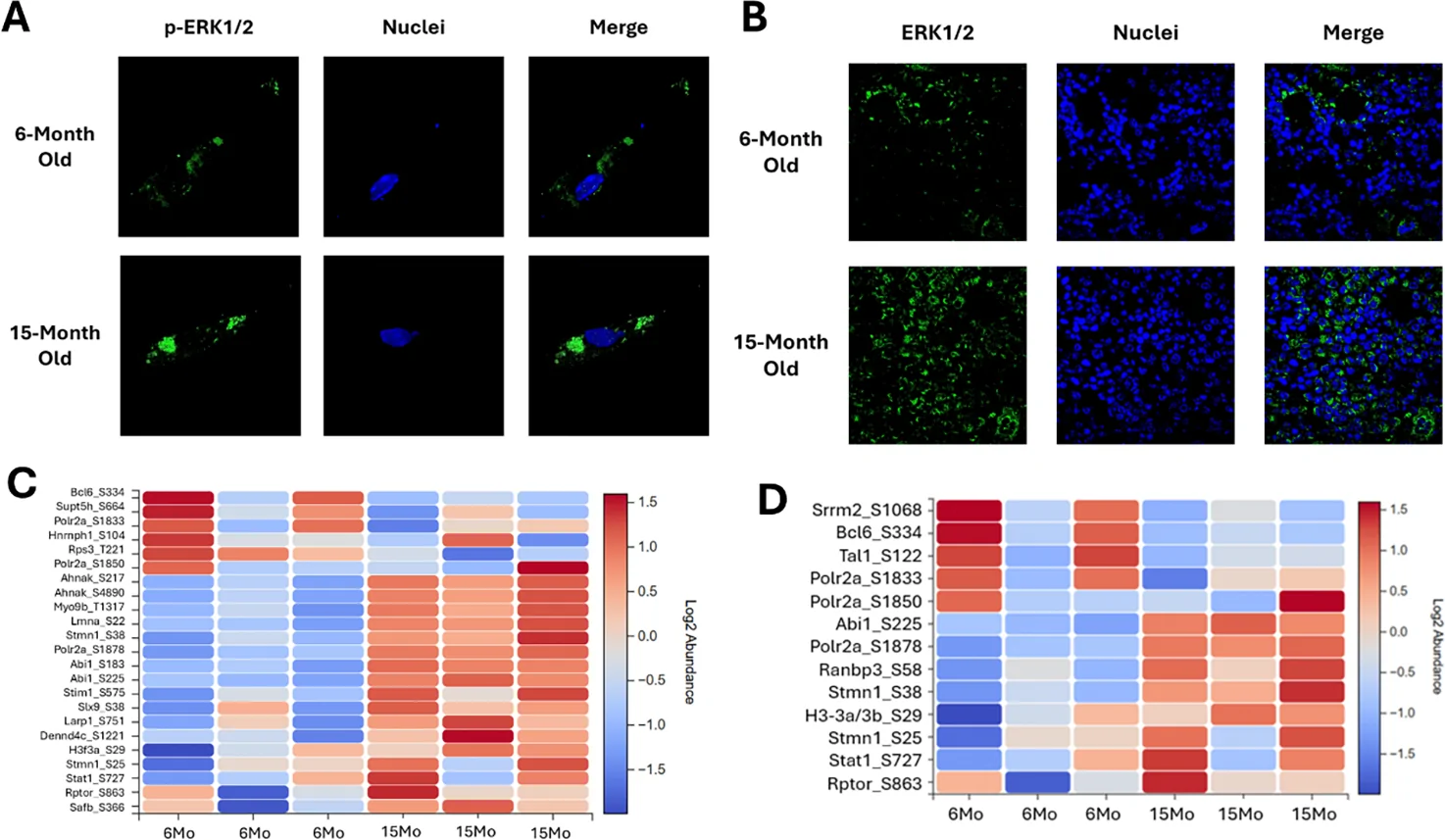
ERK1/2 activity increases with age in BMSC. (**A**) Fluorescent immunostaining for p-ERK-1/2 showing increased activating ERK-1/2 phosphorylation in the 15-month-old BMSC. (Green = p–ERK1/2; Blue = Nuclei) (**B**) Increase in total ERK-1/2 abundance and expression in the aged BMSCs relative to the young 6-month-old BMSCs. (Green = ERK1/2; Blue = Nuclei) (**C**) Changes in the phosphorylation patterns of MAPK1 (ERK2) substrates with aging in BMSCs. (**D**) MAPK3 (ERK1) substrate phosphorylation is altered with aging in BMSC.

Bone marrow mesenchymal stem cells isolated from 15-month-old mice have increased ERK-1/2 phosphorylation compared to the cells isolated from younger mice (Figure 6A). The phosphorylation of tyrosine-204 activates ERK1/2, suggesting that with aging, ERK1/2 activity increases in BMSC. Additionally, when BMSCs within the bone marrow were stained for ERK1/2, the expression level of ERK1/2 in BMSCs was elevated with age. So, ERK1/2 sees an upregulation with regard to both its activity and abundance within the mesenchymal stem cells of bone marrow (Figure 6A,B). Additionally, the substrates of both ERK1 and ERK2 undergo substantial dysregulation in phosphorylation with aging (Figure 6C,D), further supporting the role of ERK-1/2 dysfunction in BMSC aging.

## 4. Discussion

BMSCs are a key mesenchymal stem cell population and are multi-potent in nature owing to their ability to differentiate into osteoblasts, adipocytes, and chondrocytes [26,58]. There exists an equilibrium between the adipo-osteogenic lineage commitment in BMSC, where they tend to adopt more osteogenic fate over the adipogenic one [34]. With aging, this balance between osteogenic differentiation and adipogenic differentiation is completely lost, resulting in increased adipogenesis and diminished osteogenic potential [26,32,34,59]. While aberrations of BMP2 signaling cascades and induction of cellular senescence contribute to the dysregulation in fate decision, many aspects of the intracellular molecular changes in BMSC that contribute to aging and age-associated phenotype are still unknown and unresolved [32,34].

To map molecular changes affecting the activity and abundance of proteins, as well as biological processes and signaling pathways, an integrated systems biology approach was employed by combining global phosphoproteomic and total proteomic profiling of BMSCs isolated from 6-month-old and 15-month-old C57BL/6 (B6) mice. Phosphoproteomic profiling technologies enable high-throughput and precise identification and quantification of site-specific phosphorylation events, ultimately helping to delineate the dynamics of signaling cascades within the intracellular environment [12]. Phosphorylation is one of the most abundant post-translational modifications in proteins, including signaling proteins. It often directly regulates protein activity by inducing conformational change, creating binding sites, or through other mechanisms [60]. Therefore, profiling the dynamics of protein phosphorylation can inform about key changes in protein activity. Mass spectrometry-based proteomic profiling, on the other hand, enables the system-wide identification, characterization, and quantification of protein expression in a high-throughput manner, providing key information on gene expression dynamics and regulation [61]. While conventional approaches, such as Western blot and other imaging techniques, enable us to investigate specific pathway alterations, they do not provide a systemic and pathway-wide insight into age-associated changes in BMSCs. Integrative profiling and analysis of protein abundance and phosphorylation allows for a more nuanced and explicit view of the activity of key signaling modules and molecular processes within the intracellular environment. This study combines phosphoproteomic and proteomic techniques with robust systems biology and bioinformatics analysis to provide a highly explicit view and novel insights into the molecular alterations that drive BMSC aging for the very first time.

As the model for studying aging in BMSCs, BMSCs from 6-month-old and 15-month-old C57BL/6 (B6) mice were used, which represented the younger and older BMSC populations, respectively. The 15-month-old C57BL/6 (B6) mice exhibit many properties of human bone aging, such as a substantial decline in bone volume/tissue volume (BV/TV) [62]. The extent of decline in bone mass and bone volume in the 15-month-old mice is as pronounced as the 20-month-old mice [62]. Moreover, the BMSCs isolated from both the 15-month-old and 20-month-old mice show a similar degree of molecular changes with regard to BMPR1A and CK2α overexpression, which are key proteins involved in BMP2 signaling, and imply a disruption of this key osteogenic signaling cascade with aging [62]. Additionally, BMSCs of both these ages have lowered osteogenic potential and show quite a similar extent of non-responsiveness to BMP2 stimulation [62]. Therefore, the BMSCs from the 15-month-old C57BL/6 (B6) mice were used as a model for aging, instead of a much older model.

Comparative proteomic and phosphoproteomic profiling of young and aged murine BMSCs reveals a significant and systemic change in protein abundance and phosphorylation (hence activity) with aging. More than 500 proteins underwent changes in abundance and/or phosphorylation due to aging in BMSC, implying a systemwide molecular reprogramming underlying the age-associated cellular dysfunctions. Additionally, age-specific proteomic and phosphoproteomic signatures were also quite distinct, suggesting a shift in protein activity with aging. Such age-associated alterations in the proteome were also observed in mesenchymal stem cells of bone marrow in humans [63]. Interestingly, many of the proteins with age-associated alterations were involved in cellular metabolism (metabolic enzymes and transporters, Figures 1F and 3E), implying a metabolic reprogramming in aged BMSCs. This finding is also supported by the functional enrichment analysis, which reveals metabolic processes like lipid and fatty acid metabolism, the TCA cycle, as well as amino acid metabolism to be affected by aging (Figure 5A–C). In alignment with the findings, prior studies also suggest that mitochondrial dysfunctions can promote senescence, and hence aging, in mesenchymal stem cells of bone marrow [64]. This metabolic reprogramming may mediate the shift in the adipo-osteogenic lineage commitment in BMSCs with aging. Recent studies suggest that metabolic signaling, through the epigenetic regulation of gene expression, can serve as a key mediator of cell fate decisions [65]. Additionally, the metabolic state between undifferentiated, osteogenic-lineage committed, and adipogenic-lineage committed BMSCs also differs greatly, further supporting the concept.

A shift in kinase signaling and kinase-substrate phosphorylation dynamics accompanies metabolic reprogramming in aged BMSCs. Many of the kinases underwent significant changes in their phosphorylation and abundance, which is also reflected in the quantitative alterations in the kinase-substrate network, as many of their substrates also saw differential phosphorylation between the two age groups. For predicting the upstream kinases, three different techniques were employed: Kinase-Substrate Enrichment Analysis (KSEA), Robust Kinase Activity Inference (ROKAI_XPLORER), and Kinase Activity and Inference Dashboard (KINAID) [39–41]. The KSEA tool within the Phosphomatics web server provides a greater coverage of the kinase-substrate relationship library by combining PhosphositePlus, SIGNOR 2.0, and the kinome study by Sugyama et al. [38–41,44]. The KINAID tool, on the other hand, uses a sequence motif and orthology-based approach to predict the kinase-substrate relationships, providing a broader coverage and increased sensitivity to mutations within the phosphorylated sequence motifs [41]. ROKAI-XPLORER combines the resourcefulness of PhosphositePlus and the robustness of the NetworKIN algorithm to predict kinase activity [40]. Each tool brings a unique strength through distinct methodologies employed in predicting kinase activity from phosphorylation abundance data, and their integrative application provides a very comprehensive, nuanced, and robust insight into the modulation of kinase activity with aging.

Two key kinases predicted by enrichment analysis to have an elevation in their activity with aging are ERK1/2 (also known as Mapk3 and Mapk1). Fluorescent immunostaining of BMSCs ultimately shows that both phosphorylation (Tyrosine phosphorylation, which activates ERK1/2) and expression levels of ERK1/2 increase with age, supporting the bioinformatic analysis. These findings are further supported by the changes in the phosphorylation level of their substrates, including Polr2a, Lmna, Ranbp3, and Ahnak. These substrates are involved in diverse processes like transcription, nuclear transport, and nuclear structure maintenance. Interestingly, many of these substrates are localized to the nucleus and are directly or indirectly involved in gene expression regulation. Therefore, increased ERK1/2 activity in the aged BMSCs might promote adipogenic fate by influencing gene expression. Moreover, ERK1/2 is also known to be an activator of the NF-κB axis [66]. The phosphorylated and activated ERK1/2 can phosphorylate the repressor of NF-κB, called IκBα, promoting its dissociation from NF-κB [66]. NF-κB can then translocate to the nucleus to mediate its transcriptional regulatory functions. NF-κB is also known to inhibit osteogenic differentiation in BMSCs and other osteoprogenitors [67]. Therefore, it can be hypothesized that age-associated increased activation of ERK1/2 suppresses osteogenic lineage commitment by facilitating the nuclear translocation of NF-κB, providing a model for BMSC aging and associated shift in fate decision. This model is further supported by experiments where ERK1/2 inhibition increased the osteogenic differentiation in BMSC [68]. This model is well supported by discovery-level omics data and prior studies. However, experimental validation and functional connection between ERK1/2, NF-κB, and BMSC fate are still lacking. Thus, this model would require further investigation. Additionally, Mtor activity is also predicted to increase in the aged BMSCs, and prior studies suggest that higher Mtor activity can promote cellular senescence and mesenchymal stem cell dysfunction [64].

Alterations in the transcriptional landscapes of BMSCs due to aging are reflected by changes in the activity/expression of transcription factors as well as epigenetic modulators. In addition, transcription factors enriched in the upstream regulatory element of the mis-expressed genes are also found to have dysregulation in their activity. The activity change is either due to phosphorylation or expression level changes. Based on discovery-level phosphoproteomic profiling in young and aged BMSCs, Lrrfip1 was found to have increased serine phosphorylation. Besides, Ruvbl1 and Ruvbl2 were also significantly (adjusted *p*-value < 0.05) downregulated in the aged BMSCs based on proteomic analysis. However, these findings require further experimental validation through immunostaining and Western blot. Along with transcriptional reprogramming, a substantial alteration is seen in the abundance and phosphorylation of RNA-binding proteins, many of which are involved in transport mechanisms. PPI network analysis also identified a highly interconnected functional module involved in nucleocytoplasmic transport (Figure S3). These proteins either facilitate RNA transport by binding to it (Ranbp2, Srrm1, Rbm8a) or are components of the nuclear pore complex (Tpr). This infers a key role for the RNA transport process in the post-transcriptional gene regulatory mechanisms in BMSCs with aging. Even though prior studies underline the role of epigenetic modification and transcriptional changes in BMSC aging, the role of RNA transport mechanisms and RNA-binding proteins remains elusive [64,69]. Besides, there are substantial alterations in the proteolytic machinery, including proteosome subunits, in the aged BMSCs, suggesting disruption in proteostasis. Loss of proteostasis can contribute to aging, senescence, and deregulated fate decisions by the buildup of proteins and by the degradation of certain differentiation-associated proteins. Deregulation of proteostasis is also a known hallmark of mesenchymal stem cell aging, which is primarily caused by endoplasmic reticulum stress [64].

One of the most pronounced biological processes that has been found to be affected due to aging is the endocytic and vesicular transport mechanisms. Both over-representation analysis and network analysis identified the process to be implicated in BMSC aging. Prior studies on aged BMSCs show that inhibition of the endocytic mechanisms can resensitize the cells to osteogenic BMP2 stimulation and restore BMP2 signaling and osteogenic potential [32]. Past research also proposes that misexpression and mislocalization of the BMP receptor are causes for age-associated defects in BMSC [32]. This study underlies the role of clathrin-mediated endocytosis mechanisms in the receptor mislocalization and resulting aberrations in BMP2 signaling and osteogenic potential in aged BMSCs. Our model proposes that BMP receptors are mislocalized from the caveolin-rich membrane domain to the clathrin-rich domains, facilitating their endocytosis into clathrin-coated vesicles. Due to changes in the activity and expression of vesicular transport proteins, these endosomes are not shuttled back to the membrane. Consequently, the receptors are sequestered from the membrane domain where they can bind to BMP2 homodimers and transduce a signal to promote osteogenic differentiation. However, further functional analysis is necessary to confirm and validate this model, illustrating age-associated defects in BMSCs. Nevertheless, this study provides key insights into some of the mechanisms that contribute to aging and age-associated shifts in cell fate decisions in BMSCs. The signaling proteins, transcription factors, and cellular processes identified in the study as key mediators of aging in BMSCs can be targeted therapeutically to restore osteogenic potential in aged BMSCs and ultimately treat age-associated bone loss and osteoporosis.

## Supplementary Material

Supplemetal data

**Supplementary Materials:** The following supporting information can be downloaded at: https://www.mdpi.com/article/10.3390/app16083779/s1. Figure S1: Phosphosite phosphorylation dynamics in bone marrow mesenchymal stem cells (BMSCs) with aging. Figure S2: Heatmap depicting the deregulated transcription factors and their regulated genes/proteins in aged bone marrow mesenchymal stem cells. Figure S3: PCA Analysis and Integrated PPI Network Analysis.

## Data Availability

Any data or material that supports the findings of this study can be made available by the corresponding author upon request.
