# Supplementary material for: Aging Reprograms the Signaling, Metabolic, and Gene Regulatory Dynamics in Murine Bone Marrow-Derived Mesenchymal Stem Cells
Source: Appl Sci (Basel). Author manuscript; Available in PMC 2026 Sep 5. (PMC13544354; doi:10.3390/app16083779)
Supplement: Supplementary file 1 [file NIHMS2201972-supplement-Supplemetal_data.pdf]

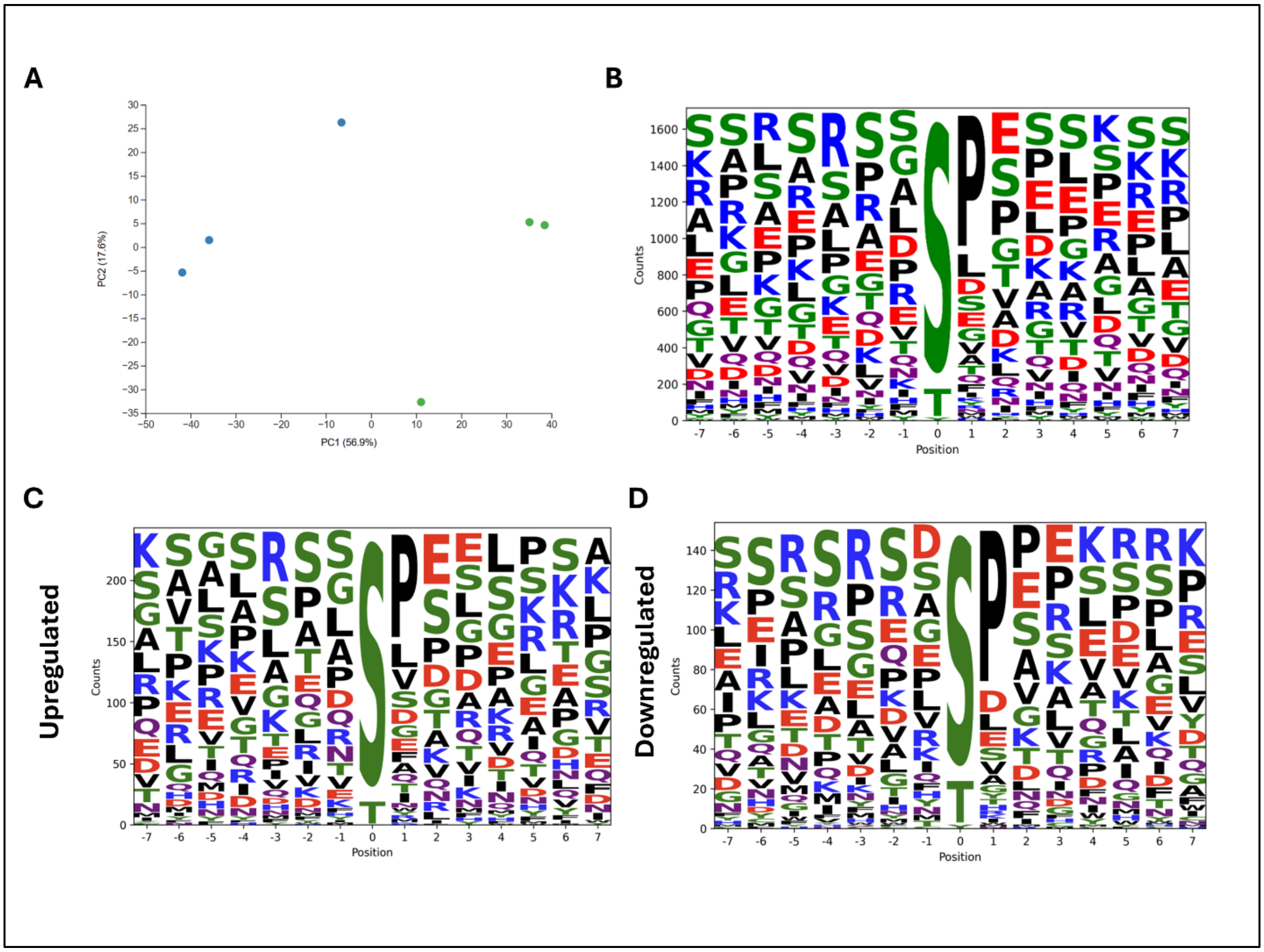

Figure S1: Phosphosite phosphorylation dynamics in bone marrow mesenchymal stem cells (BMSCs) with aging. A. Principal Component Analysis of phosphoprotein abundance data. **B.** Sequence logo for all the sequenced phosphosites in the phosphoproteins. **C.** Phosphosites that underwent upregulation in phosphorylation with age in BMSCs. **D.** Phosphosites that underwent downregulation in phosphorylation with age in BMSCs.

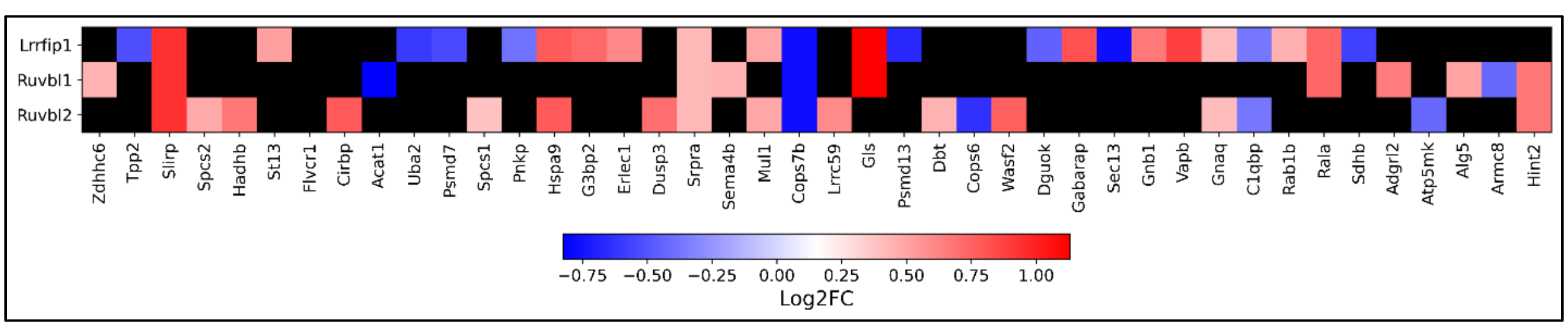

Figure S2: Heatmap depicting the deregulated transcription factors and their regulated genes/proteins in aged bone marrow mesenchymal stem cells. Transcriptional regulators, Lrrfip1, Ruvbl1, and Ruvbl2, and their target genes undergo significant alterations in expression with aging in mouse BMSCs. (Red = Upregulated; Blue = Downregulated; Black = Does not regulate).

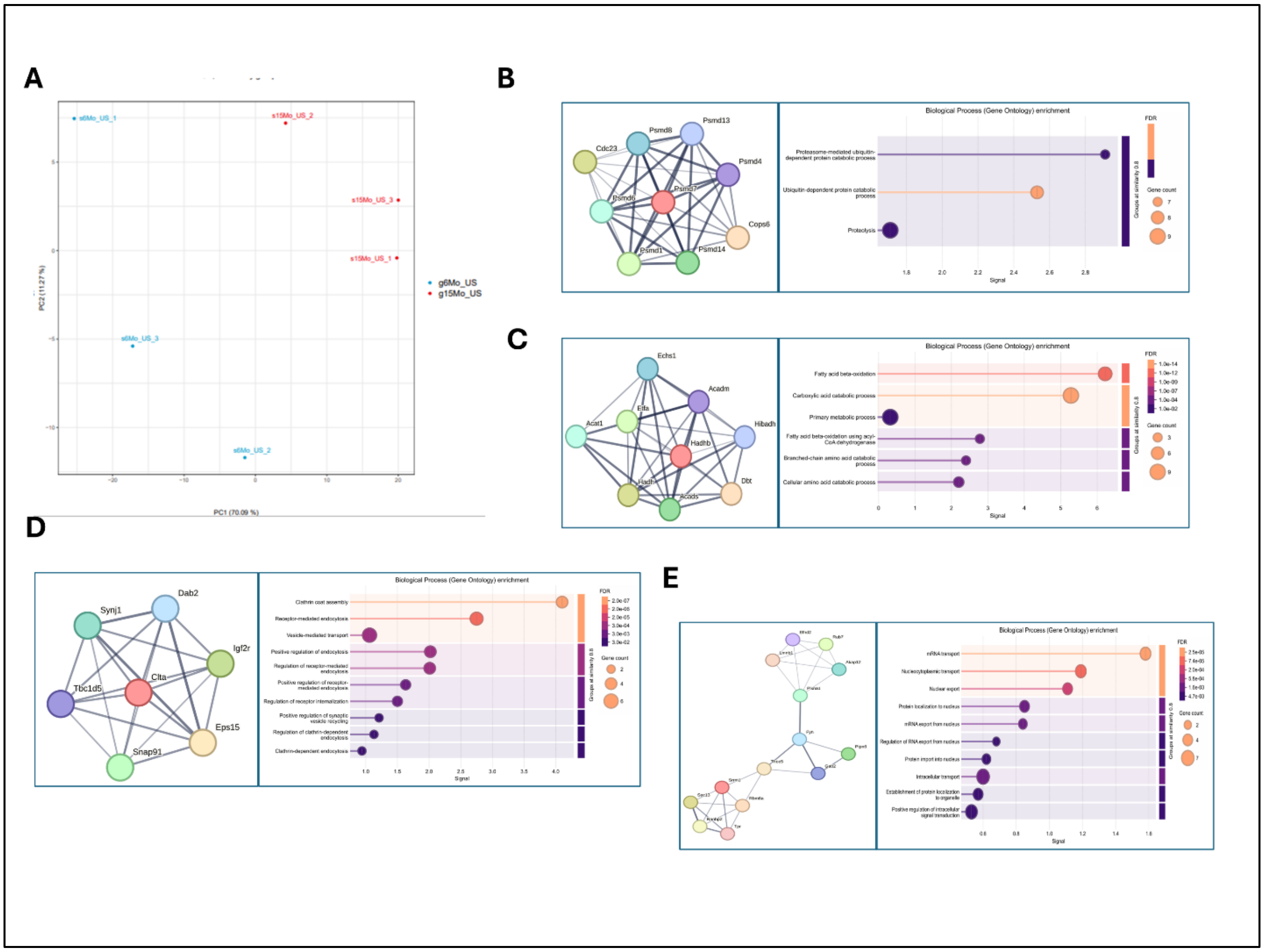

Figure S3: PCA Analysis and Integrated PPI Network Analysis. A. Principal Component Analysis (PCA) of protein expression data. **B.** The highest-ranked cluster in the Protein-protein Interaction (PPI) network and its biological function. **C.** The protein interaction module is involved in fatty acid metabolism. **D.** Functional module of highly interconnected proteins that function in endocytic and vesicular transport processes. **E.** Cluster of proteins that mediate and regulate nucleocytoplasmic transport.
